# Recent Progress of Stem Cell Therapy in Cancer Treatment: Molecular Mechanisms and Potential Applications

**DOI:** 10.3390/cells9030563

**Published:** 2020-02-28

**Authors:** Dinh-Toi Chu, Tiep Tien Nguyen, Nguyen Le Bao Tien, Dang-Khoa Tran, Jee-Heon Jeong, Pham Gia Anh, Vo Van Thanh, Dang Tien Truong, Thien Chu Dinh

**Affiliations:** 1 Department of Human and Animal Physiology, Faculty of Biology, Hanoi National University of Education, Hanoi 100000, Vietnam; 2 College of Pharmacy, Yeungnam University, 280 Daehak-ro, Gyeongsan-si, Gyeongbuk-do 38541, Korea; tientiephup@gmail.com (T.T.N.); jeeheon@yu.ac.kr (J.-H.J.); 3 Institute of Orthopaedics and Trauma Surgery, Viet Duc Hospital, Hanoi 100000, Vietnam; bstiencsvd@gmail.com (N.L.B.T.); thanhhmu@gmail.com (V.V.T.); 4 Department of Anatomy, University of Medicine Pham Ngoc Thach, Ho Chi Minh City 700000, Vietnam; khoatrandr@gmail.com; 5 Oncology Department, Viet Duc Hospital, Hanoi 100000, Vietnam; phamgiaanh@gmail.com; 6 Department of Surgery, Hanoi Medical University, Hanoi 100000, Vietnam; 7 Department of Anatomy, Vietnam Military Medical University, Hanoi 100000, Vietnam; truongdtvmmu@gmail.com; 8 Institute for Research and Development, Duy Tan University, Danang 550000, Vietnam

**Keywords:** stem cells, cancer treatment, mechanisms, application

## Abstract

The insufficient and unspecific target of traditional therapeutic approaches in cancer treatment often leads to therapy resistance and cancer recurrence. Over the past decades, accumulating discoveries about stem cell biology have provided new potential approaches to cure cancer patients. Stem cells possess unique biological actions, including self-renewal, directional migration, differentiation, and modulatory effects on other cells, which can be utilized as regenerative medicine, therapeutic carriers, drug targeting, and generation of immune cells. In this review, we emphasize the mechanisms underlying the use of various types of stem cells in cancer treatment. In addition, we summarize recent progress in the clinical applications of stem cells, as well as common risks of this therapy. We finally give general directions for future studies, aiming to improve overall outcomes in the fight against cancer.

## 1. Introduction

Cancer is the most dangerous disease by causing millions of deaths worldwide [1]. Despite of rapid advancement in research of diagnostics and therapeutics, the death rate by cancer only declined ~1.5% annually in the period of 2006–2015 in United States [1]. Comprehensive knowledge about cancer biology would allow scientists to design better therapeutic systems.

The type of treatment depends on cancer type and progression, and treatment purpose. Surgery is the first option for direct removal of solid tumors located in one area. Radiotherapy can kill tumors by damaging cancer cell DNA. Chemotherapy, with the use of very toxic drugs, helps slow down or stop tumor growth. Immunotherapy, including the use of monoclonal antibodies, checkpoint inhibitors, cancer vaccines, and adoptive cell transfer, now becomes an important curative for cancer with significantly improved clinical outcomes. However, the huge disadvantages of current therapies are insufficient and unspecific target of therapeutics to tumor sites except for effector chimeric antigen receptor (CAR)-cells, resulting in suboptimal efficacy, therapy resistance and subsequent tumor recurrence [2,3]. In addition, many adverse events related to off-target effects of therapeutic drugs and immune responses have been observed [2,3,4].

Meanwhile, stem cell therapy, which involves all procedures using stem cells, has provided a hopeful option in the fight against cancer. It could improve the therapeutic efficacy of other therapies due to its enhanced target on tumors, thereby reducing off-target events. Numerous stem cell-based strategies have now been under investigation in preclinical trials, and they show both great promises and challenges for cancer treatment [5]. Therefore, further evaluation is needed to make them feasible for upcoming clinical trials. The aims of this study are to provide an overview of the type of stem cells and mechanisms underlying the action of stem cells in cancer treatment. In addition, we will update recent advances as well as side effects related to this therapy. General future directions will also be given as a part of this review.

## 2. Type of Stem Cells for Cancer Treatment

Stem cells from different sources exhibit different capacities of proliferation, migration, and differentiation, which determine their application in anti-tumor therapy.

### 2.1. Pluripotent Stem Cells (PSCs)

Embryonic stem cells (ESCs) isolated from the undifferentiated inner mass cells of embryo possess the ability to give rise to all types of cells except those in the placenta. However, the applications of ESCs for clinical trials are restricted due to ethical considerations. In 2006, the invention of Yamanaka factors to induce pluripotent stem cells (iPSCs) from somatic cells in culture marked a breakthrough in cell biology [6]. These iPSCs share the same characteristics with ESCs while removing ethical concerns from embryo destruction. Till now, both hESCs and iPSCs are important sources for the induction of effector T- and NK cells [7,8,9,10], and for the production of anti-cancer vaccines [11,12], which will be discussed later in this review.

### 2.2. Adult Stem Cells (ASCs)

ASCs can give rise to many specialized cell types of the tissue and organ. In this group, hematopoietic stem cells (HSCs), mesenchymal stem cells (MSCs), and neural stem cells (NSCs) are often utilized in cancer treatment.

HSCs, located in bone marrow, can form all mature blood cells in the body. Till now, the infusion of HSCs derived from cord blood is the only procedure of stem cells that were approved by the FDA to treat multiple myeloma, leukemia, and some kinds of blood system disorders [13].

MSCs are found in many tissues and organs, playing important roles on tissue repair and regeneration. They can rapidly proliferate and generate several specialized cell types in vitro, such as osteocytes, adipocytes, and chondrocytes. MSCs possess unique biological properties and have been extensively used to support other therapies or to deliver therapeutic agents in treating a variety of cancers [14,15].

NSCs, originally present in the central nervous system, can self-renew and generate new neurons and glial cells. They have been broadly tested to treat both primary and metastatic breast, lung, and prostate cancers in murine models [16,17,18].

### 2.3. Cancer Stem Cells (CSCs)

CSCs, so-called stem-like cells or immature progenitors of tumor cells or tumor-initiating cells, are generated by epigenetic mutations in normal stem cells or in precursor/progenitor cells. CSCs are found within tumor tissues, playing important roles in cancer growth, metastasis, and recurrence [19]. Therefore, targeting CSCs could provide a promise to treat various types of solid tumors.

## 3. Mechanisms Underlying the Action of Stem Cells in Cancer

### 3.1. Homing to Bone Marrow

In aggressive tumors, blood-forming cells and leukocytes are absolutely or partially damaged by the use of very high doses of chemotherapy for removal of all cancer cells [13]. In those conditions, patients need to receive the intravenous infusion of autologous or allogeneic HSCs. These HSCs are supposed to exert a homing process that leads to their rapid migration into defined stem cell niches in bone marrow (BM). After encountering BM, the transplanted HSCs undergo the engraftment process prior to giving rise to specialized blood cells.

The molecular mechanism behind the HSC homing process is mainly dependent on the active interaction between stem cell CXCR4 receptor with gradient SDF-1 secreted from cells lining the BM stem cell niches. Other molecular signalings involve ceramide-1 phosphate, sphingosine-1-phosphate [20,21,22], extracellular ATP or UTP [23], and Ca^2+^ and H^+^ ions [24,25]. Furthermore, the transmigration of HSCs through blood vessels requires their interaction with endothelial cells via LFA-1, VLA-4/5, CD44, and the secretion of matrix degradable enzyme MMP-2/9 [26].

### 3.2. Tumor-Tropic Effect

Tumor microenvironment where extracellular matrix (ECM) and secreted paracrine factors are deposited defines tumor growth and invasion by attracting the directional migration of various types of cells, such as endothelial cells, infiltrating immune cells, and MSCs. Tumors are considered chronic wound tissue with sustained hypoxia, inflammation, and oxidative stress events but never heal [27,28,29]. Therefore, the migration of MSCs toward tumor microenvironment is supposed to be similar to their migration to injured or ischemic sites. In fact, both tumor cells and tumor-associated immune cells can involve this process through the secretion of various chemoattractant factors [30,31]. For example, CXCL16, SDF-1, CCL-25, and IL-6 secreted from prostate cancer, osteosarcoma, multiple myeloma, and breast cancer cells attract migration of MSCs toward tumor microenvironment, respectively [32,33,34,35]. Pro-inflammatory cytokines (TNF-α and IL-1β) induced by tumor-associated immune cells play crucial roles in the migration and differentiation of MSCs within the tumor [36,37]. In tumor sites, MSCs can differentiate into endothelial cells or myofibroblasts, which contributes to tumor stromal development.

### 3.3. Paracrine Factor Secretion and Differentiation Capacity

Growing pieces of evidence have demonstrated that stem cells do their functions by releasing various paracrine factors, including extracellular vesicles (EVs) and soluble materials [38], which may affect tumor survival, progression, and metastasis. Among three types of EVs, nano-sized exosomes exhibit as a crucial regulator of cell-cell interaction. They are produced by the exocytosis process of the intracellular multivesicular body, containing various biological molecules, such as proteins, mRNAs, and miRNAs [38]. Once released from MSCs, exosomes can target tumor niche where they are internalized into cancer cells via endocytosis or receptor-ligand interaction, then release their cargo within receiver cells [38]. Also, stem cells can respond to stimulus to produce numerous soluble factors [39]. For instance, MSCs can release immunosuppressive factors (TGF-β1, IDO, iNOS, HLA-G), anti-inflammatory factors (TSG-6, IL-1RA, PGE-2), anti-apoptotic factor (STC-1), and angiogenic factors (VEGF, HO-1, IGF-1) [39].

Moreover, the differentiation capacity of stem cells is highly important for cancer treatment. For example, transplanted HSCs can give rise to all blood cell types. Therefore, the number and quality of engrafted HSCs would affect clinical outcomes and the recovery of blood system [40]. Likewise, NSCs can replace the injured neuron and glial cells in brain cancer. ESCs and iPSCs can serve as cell sources for the induction of effector immune cells to specifically target a tumor.

### 3.4. Signaling in CSCs

**Figure 1:**
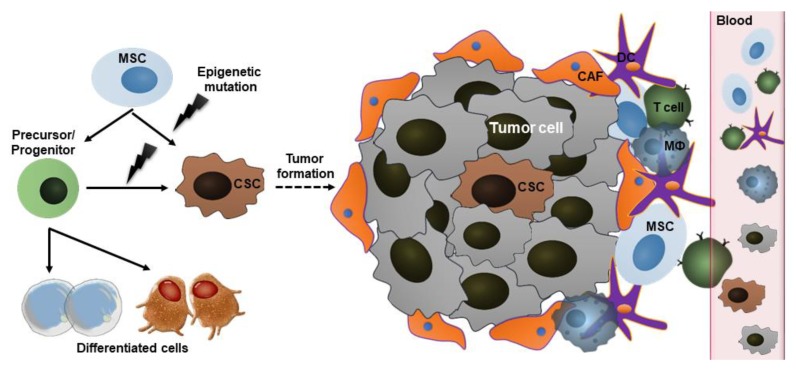
Tumor formation and its complex microenvironment. Epigenetic mutations in normal stem cells or precursor/progenitor cells lead to generation of CSCs, which play crucial roles in tumor stemness, initiation, maintenance, and metastasis. Tumor niche contains not only tumor cells and CSCs but also cancer-associated immune cells (T-cells, macrophages (MΦ), dendritic cells (DC)), cancer-associated fibroblasts (CAF), and other cells. In addition, MSCs are recruited to tumor by chemoattractant factors secreted from all those cells (CXCL16, SDF-1, CCL-25, and IL-6, TNF-α, and IL-1β), playing crucial roles in tumor progression.

It is well-established that molecular signaling pathways, including Notch, Hedgehog, Wnt/β-catenin, PI3K/PTEN, JAK/STAT, and NF-κB, regulate normal stem cell proliferation. The persistent change in those signaling pathways will bring about the formation of CSCs and subsequent cancer cells [41]. CSCs have a high capability of self-renewal and differentiation to aid tumor growth, recurrence and metastasis (Figure 1) [42,43]. Also, these cells are responsible for conventional therapy resistance of tumors [19,44,45]. Therefore, research on CSCs is highly important to develop an effective therapy for cancer treatment.

CSCs are reported to exist in a variety of tumor types (leukemia, brain, breast, lung, and gastrointestinal cancers), and are typically isolated and identified using various strategies including surface protein markers and metabolic/functional properties [46,47]. Origin from normal stem cells, key characteristics and biological pathways in CSCs share some similarities with the former. Some surface markers, such as CD133 (Prominin-1, HSC marker), CD44, and CD24, Lgr5, and EpCAM, are often used to characterize CSCs from highly heterogeneous cell types in tumors, though they can be also expressed in normal tissues [46]. CD133^+^ cells were successfully purified from the brain, lung, colorectal, liver, and gastric cancers, which exhibited higher self-renewal capacity and the initiation of the original tumor in vivo. Likewise, CD44, in combination with CD133 or CD24, is widely used to determine the CSC population in various tumor types. In the NOD/SCID mice model of breast cancer, CD44^+^CD24^−/low^ cells exist in a very small population but possess the ability to aid tumor growth and even form complete original tumor tissue [48].

CSCs are furtherly characterized by evaluating their metabolic/functional activities. In this aspect, aldehyde dehydrogenase (ALDH) enzymes, which catalyze aldehyde oxidation in various physiological substrates and toxins, are often measured. High ALDH levels, using a flow cytometry-based ALDEFLUOR assay, are found in the cells with high metabolic activities, such as normal stem cells and CSCs. ALDH^high^CD44^+^CD24^−^ and ALDH^high^CD44^+^CD133^+^ cells found in breast cancer exhibited greater tumorigenicity than the corresponding low/negative populations [49].

In addition, CSCs could be identified with high glycolic activity, slow cell division, enhanced therapy resistance, and enhanced immune resistance [46]. Especially, tumor-associated antigens (TAAs) in CSCs including cancer/testis antigens and oncofetal antigens which are typically expressed only in germ cells and embryonic development, could be served as good targeting molecules for immunotherapy.

## 4. The Potential Applications of Stem Cell Therapy in Cancer

**Figure 2:**
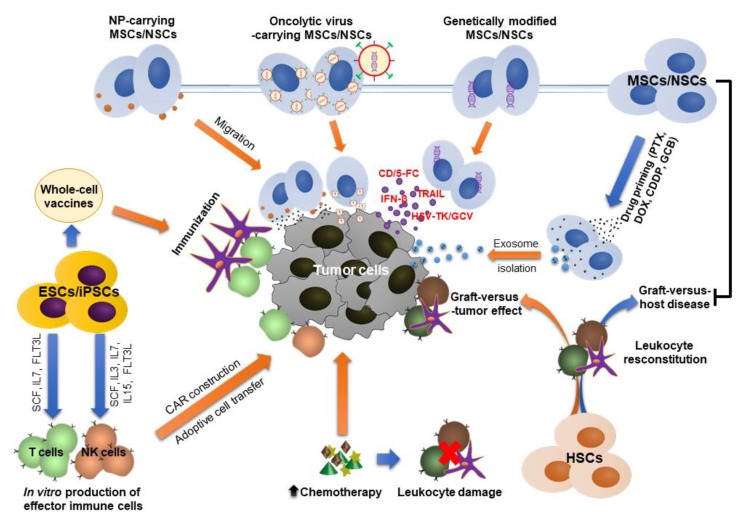
Strategies for the application of stem cell therapy in the treatment of cancer. (1) HSC transplantation has been used for the reconstitution of blood-forming cells and leukocytes after heavy chemotherapy or radiotherapy. (2) ESCs and iPSCs can be used for the production of effector immune cells that are then CAR constructed for adoptive cell transfer technology. In addition, ESCs and iPSCs can be potential sources for the production of anti-cancer vaccines. (3) MSCs/NSCs are effective to deliver genes, NPs, and OVs to tumor niche due to their intrinsic tumor tropism. In addition, exosomes extracted from the culture of drug-priming MSCs/NSCs can be used to target the drugs to tumor sites. Moreover, MSCs are capable of reducing GVHD in HSC transplantation.

Various strategies have been developed for cancer treatment using stem cell therapy, including HSC transplantation, MSC infusion for post-cancer treatment, stem cells for therapeutic carriers, generation of immune effector cells, and vaccine production (Figure 2).

### 4.1. HSC Transplantation

HSC transplantation has been primarily used as a standard procedure for the treatment of multiple myeloma, leukemia, and lymphomas after rounds of high-dose radiotherapy or chemotherapy [13] (clinicaltrials.gov). In addition, this procedure is now widely investigated in clinical trials, in combination with chemotherapy or immunotherapy, to treat other kinds of cancer, such as brain tumors (NCT00528437), neuroblastoma, sarcomas (NCT01807468), and breast cancer (NCT00003927). However, the occurrence of graft-versus-host-disease (GVHD) when using allogeneic sources of HSCs remains a challenge, which is often treated with immunosuppressive drugs with less effectiveness and serious side effects [50].

### 4.2. MSC Transplantation After Cancer Treatment

Treatment of aggressive cancers that involves invasive tumor removal and high-dose therapy often leads to damage in normal tissues and hematopoietic system. Clear evidence has shown the infusion of MSCs helps maintaining the undifferentiated state and proliferation of HSCs, thereby enhancing the overall outcome of the treatment [51,52,53]. In addition, MSCs with immunomodulatory effects could effectively reduce strong immune responses in patients with refractory GVHD. Recent clinical trials have shown promising outcomes with no related adverse effects following the co-transplantation of MSCs and HSCs (Table 1). One ongoing multi-center trial (NCT02923375) is testing the safety, tolerability, and efficacy of mesenchymoangioblast-derived MSC infusion in adults who have steroid-resistant GVHD. MSCs are also found to facilitate the recovery of injured organs and could enable body tolerance to high-dose chemotherapy that is to improve tumor-killing effects [39].

### 4.3. Stem Cells as Potential Therapeutic Carriers

The rationales for using stem cell carriers in cancer treatment are to: (i) protect therapeutic agents from rapidly biological degradation, (ii) reduce systemic side effects and (iii) increase local levels of therapeutics due to intrinsic tumor-targeting effect of stem cells. The anti-tumor efficacy of this system relies on the number of stem cells localized into tumor microenvironment.

#### 4.3.1. Genetically Modified Stem Cells

In this approach, stem cells, including MSCs and NSCs, are virally transduced to enhance the expression and secretion of soluble factors which are prodrug-converting enzymes or tumor-toxic cytokines/chemokines [59]. The former system is well-known for its name “suicide gene therapy” or “gene directed enzyme prodrug therapy (GDEPT)” [60]. Enzyme releasing from viable stem cells can covert prodrugs to active molecules that exhibit more toxic to cancer cells. For example, 5-fluorocytosine (5-FC) is effectively converted into tumor-toxic 5-fluororacil (5-FU) following infusion of cytosine deaminase (CD)-expressing MSCs or NSCs [17,61,62]. Likewise, irinotecan, a less potent prodrug, can be metabolized into a 1000-fold more toxic compound (SN-38) in the presence of carboxylesterase (CE). In the neuroblastoma mice model, the tumor grafts were found more sensitive to co-administration of CE-expressing NSCs and irinotecan, in comparison to single-drug treatment [63,64]. Till now, several phase I/II clinical trials on the GDEPT system have been tested (Table 2). In the patients with recurrent high-grade gliomas, CD-expressing human NSCs were directly injected into the brain whereas 5-FC was orally administered (NCT01172964, completed). In addition, leucovorin was additionally used for enhancing the tumor-killing effect of 5-FU (NCT02015819, ongoing). Moreover, autologous MSCs that expressed Herpes simplex virus thymidine kinase (HSV-TK) enzyme were utilized to treat human advanced gastrointestinal cancer (EudraCT number 2012-003741-15). HSV-TK converts ganciclovir from monophosphorylate to triphosphate form with a more potent cytotoxic effect. The combination of transduced MSCs and the prodrugs were found safe and tolerable and could enable the stabilization of cancer status [65,66].

Additionally, stem cells can genetically express secretable cytokines/chemokines. Tumor-toxic TNF-α-related apoptosis-inducing ligand (TRAIL) exhibits short half-life after its systemic delivery. Interestingly, NSCs, used as a gene carrier to continuously release TRAIL, have been proven to reduce the brain tumor metastasis and improve the survival of treated mice [67]. This system has been employed to treat human lung adenocarcinoma in a clinical trial (NCT03298763, recruiting). Co-expression of the suicide gene (i.e., CD) and cytotoxic gene (i.e., IFN-β, prevents tumor growth and prompts their apoptosis) in stem cells shows a promise to enhance treatment efficacy [68,69].

#### 4.3.2. Nanoparticles (NPs)-Carrying Stem Cells

NPs have been utilized for the carriage of anti-cancer drugs for a long time. However, their rapid excretion from the body, lack of targeting effect, and uncontrollable uptake by normal cells are the main drawbacks [70]. It is interesting that stem cells can act as effective vehicles for carrying NPs to tumor sites. In this approach, NPs could be loaded into cells or conjugated onto the cell surface.

Internalization of NPs is via passive uptake or active endocytosis, which depends on the size, surface characteristics and concentration of NPs, as well as incubation time [71]. The main concerns are toxicity potentials to the cell carrier and drug loading control. In addition, rapid NP exocytosis by the cells may lead to an uncontrollable release of therapeutic drugs to non-targeted areas. Roger et al. presented the effective internalization of both lipid and PLA NPs into MSCs without affecting cell viability and functionality [72]. In the mice model of human glioma, MSCs carried these NPs toward brain tumors after their direct tumoral injection [72]. In another study, intravenous injection of MSCs loaded with paclitaxel-loaded NPs (PTX-NPs) exhibited more NP localization and created local drug depots in the developed orthotopic lung tumor in mice [73]. Interestingly, these NP-loaded MSCs significantly inhibited tumor growth and enhanced survival of mice, though total doses of PTX-NPs were much lower than that of PTX solution or PTX-NPs alone [73]. It was found that MSCs were first entrapped in the lung parenchyma tissue and then migrated toward tumor sites due to their tumor-tropic effect [74,75]. The approach that increases the uptake of NPs by cells may improve therapeutic outcomes. For example, transactivator of transcription (TAT) peptide was utilized for enhanced internalization of PLGA NPs into MSCs [76].

Meanwhile, NPs can be targeted toward tumors by anchoring on stem cells, often via interaction with amine or thiol functional groups of cell surface [77,78,79,80]. Recently, Layek et al. reported that the loading efficiency and retention of NPs on cells could be improved by conjugating cyclooctyne-modified NPs with azide-functionalized MSCs to form a stable triazole at ambient conditions [81]. By this method, the content of PTX was ~48 pg per cell, much higher than that reported by other techniques (~1–20 pg per cell), while maintaining cell phenotype. In another study, Suryaprakash et al. engineered hybrid spheroids of TRAIL-expressing MSCs and drug-loading NPs to actively deliver combinational therapeutics for treatments of glioblastoma [82]. The intra-tumor injection of these platforms significantly improved their retention and increased the amount of delivered NPs at tumor site for enhanced anti-tumor effects [82]. Despite rapid progress in therapeutic cell engineering, there has been no clinical trial which utilizes stem cells as carriers for targeting NPs to tumors so far.

#### 4.3.3. Stem Cells as Carriers for Oncolytic Viruses (OVs)

OVs exhibit selective replication in cancer cells. It is postulated that OVs could induce tumor cell lysis, and release danger signals to activate the immune system for more effective killing of tumor cells [83]. However, naked OVs can be easily recognized by immune cells and rapidly removed from the body. Stem cells could be offered as promising carriers to protect and deliver OVs to tumor sites. For example, the human NSC line transduced with CRAd-Survivin-pk7 OV, in combination with ionizing radiation and temozolomide, could enhance cytotoxicity to glioma cells in vitro and increase the survival time of glioblastoma multiforme (GBM)-bearing mice [84]. Likewise, MSCs were found to effectively load attenuated oncolytic measles virus (OMV) and oncolytic HSV, which suppressed the growth of hepatocellular carcinoma and GBM in mice, respectively [85,86]. Interestingly, MSCs isolated from ovarian cancer patients exhibited comparable proliferation rates and good potential for OMV carriers as those from healthy donors [87]. This activity was maintained after thawing the frozen transduced MSCs, indicating their feasibility for cancer treatment.

However, the efficacy of stem cell carriers depends on their therapeutic effects as well as the relationship between vehicle cells and tumor cells. For example, both MSCs and NSCs were found to support viral replication, but a larger number of viruses could be released from NSCs. In addition, transduced NSCs exhibited more effectiveness to prolong the survival time of glioma-bearing mice [88].

#### 4.3.4. Stem Cell-Derived Exosomes as Therapeutic Carriers

Exosomes have been utilized to encapsulate various anti-cancer therapeutic materials, such as mi-RNAs, proteins, or small drugs. These natural carriers possess numerous benefits over other synthetic nanoparticulates, including unique biocompatibility, stability, high capacity for cargo loading, and enhanced internalization into tumor cells [89]. In addition, they can be easily functionalized with specific proteins or ligands on their surface to enhance targeting effect to tumor microenvironment [90,91,92].

Genetic materials, including anti-tumor mRNAs or siRNAs, were successfully packaged into stem cell-derived exosomes by traditional transfection technique. For example, Katakowski et al. collected exosomes released from miR-146b-expressing marrow stromal cells [93]. In a rat model of primary brain tumor, direct injection of these exosomes into tumors brought about a remarkable reduction in glioma xenograft growth [93]. In another study, exosomes secreted from miR-122-expressing MSCs significantly enhanced antitumor effect of sorafenib on hepatocellular carcinoma tumor model [94]. Likewise, MSC-derived exosomes efficiently delivered siRNA to bladder cancer cells for silencing polo-like kinase 1 gene [95].

Meanwhile, small molecule drugs could be encapsulated into exosomes by two approaches. First, it was found that after priming with exogenous materials, stem cells can uptake, package those agents into exosomes, and then release them to culture medium by exocytosis process. Pascucci et al. reported that exosomes extracted from paclitaxel-priming MSCs dramatically inhibited the proliferation of human pancreatic adenocarcinoma cell line [96]. In addition, these exosomes were found to suppress tumor growth of leukemia and myeloma cell lines [97,98]. Other drugs, including doxorubicin, gemcitabine, and cisplatin, have been used to prime MSCs [99]. In fact, the content of drugs in exosomes largely depends on their priming concentration, incubation time, and cell uptake mechanism [96]. Instead, therapeutic drugs could be loaded into exosomes by the post-loading method. After extraction from the culture medium of stem cells, exosomes were forced to encapsulate drugs by using extrusion, electroporation, dialysis, or saponin-assisted method [89]. This enables to load both hydrophilic and hydrophobic drugs, as well as to control the drug loading more precisely and enhance its encapsulation efficiency.

### 4.4. Stem Cell Source for Production of Immune Cells

Chimeric antigen receptor (CAR) T cells and natural killer (NK) cells have been successfully applied for anticancer immunotherapy. These clinical-grade immune cells are often harvested from the own patient, activated, genetically transduced with CAR constructs, expanded, and then reinfused to the patient [100]. However, controlling the quantity and quality of those cells for immunotherapy remains challenging, especially in patients who experienced heavy chemotherapy or in whom with higher ages. In addition, in vivo anti-tumor activity of these CAR immune cells is often limited due to their rapid differentiation into short-lived effector cells [101]. Therefore, there are requirements for the generation of CAR cells from other sources, which enables the expansion of this immunotherapy to a larger number of patients [9,102]. Human pluripotent stem cells, including iPSCs and ESCs, could offer unlimited sources for this purpose [102].

The differentiation process involves the incubation of stem cells in the growth medium containing NK cell or T cell-initiating cytokines for a month. For example, to induce NK cell differentiation, priming cytokines contain stem cell factor (SCF), IL-3, IL-7, IL-15, and fms-like tyrosine kinase receptor-3 ligand (FLT3L) [7,8]. To induce T cell differentiation, hESCs and bone marrow stromal cells (OP9) were cultured in the medium containing SCF, IL-7, and FLT3L [10,103].

Interestingly, the induction of CAR on HSCs exhibits more benefits in treating cancer. Transplanted CAR-HSCs would engraft in bone marrow and continuously generate various CAR-expressing immune cells, such as T cells, NK cells, monocytes, and neutrophils. The combination effect of these cells would result in more strong immunity to kill cancer [101].

### 4.5. Stem Cell-Based Anti-Cancer Vaccines

With undeniable roles of CSCs on tumor formation and progression, therapies that target CSCs would remarkably improve therapeutic efficacy in fighting against cancers [19,45,46]. Among various CSC-targeting approaches, the production of anti-cancer vaccines exhibits highly promising due to their high immunogenicity [45]. Anti-cancer vaccines could be raised from oncofetal peptides or CSC/ESC/iPSC-based whole-cells [12]. The production of these vaccines often involves the loading of antigens onto dendritic cells, which is then used to generate primary T-cell responses in vivo or to prime engineered T-cells in vitro for adoptive cell therapy [46]. The single use of oncofecal peptide-based vaccines cannot provide sufficient immune responses toward tumors due to tumor heterogeneity and rapid escape mechanisms [12]. Anti-cancer vaccines based on whole-cell lysates may result in better outcomes. Till now, isolation of a small population of CSCs from tumor tissue remains challenging in spite of rapid progress in the identification of these cells, which hinders the use of CSCs as a vaccine source [28].

Meanwhile, vaccines produced by ESCs/iPSCs-derived multiple antigens would offer more applications to treat cancers. Main concerns are the possibility of tetratoma formation and induction of autoimmunity [11,104,105]. In comparison to a xenogeneic source, allogeneic ESCs and autologous iPSCs-based vaccines were found more effective to prevent tumor relapse in mice, as evidenced by enhanced immune cell proliferation and cytotoxic cytokine production [104,105,106,107]. However, these vaccines should be used as prophylactic treatment rather than as a therapeutic treatment. Preformed tumors with a strong immunosuppressive microenvironment could reduce the efficacy of vaccine treatment [108]. To enhance anti-tumor immunity, combination with other therapies, such as surgery, radiation therapy, chemotherapy, immune checkpoint inhibitors, and adjuvants, is necessary [11].

## 5. Side Effects and Potential Risks of Stem Cell Therapy

### 5.1. Tumorigenesis

Normal stem cells and CSCs share their key biological signaling pathways. When the microenvironment of stem cells alters unfavorably, normal stem cells may transform into CSCs, subsequently leading to the formation of entire tumor tissue [109]. In man, endogenous stem cells are strictly monitored by other surrounding cells; hence, they get to work in the right way as normal. However, transplanted stem cells that are exposed to external conditions during culture prior to transplantation could change their genomic expression and subsequent phenotype. The longer culture time, stem cells are more likely cancer cells. It was found that 45.8% of MSCs spontaneously transformed into malignant cells after 1 longer month in culture [110]. Though this phenomenon is still controversial, it is important to note that the culture condition should be tightly controlled to minimize the negative change in stem cells.

PSCs are found more tumorigenic than ASCs. However, it should differentiate between “tetratoma” and “tetratocarcinoma” formation. The first term often implies inherent characteristics of PSCs to form “normal tumor”, whereas the second implies “abnormal human tumor”. There are many ways to prevent tumorigenesis of PSCs. For example, Katsukawa et al. treated iPSCs with a lethal dose of gamma irradiation and found a significant reduction in teratoma formation in mice [104,105].

In addition, stem cells could facilitate the growth and metastasis of existing tumors. For example, the weakly metastatic breast carcinoma cells were found to enhance their migration potency after co-transplantation with MSCs into mouse subcutaneous space [111].

### 5.2. Adverse Events in Allogeneic HSC Transplantation

Allogeneic HSC transplantation becomes an effective procedure to treat hematologic and lymphoid cancers. However, it can induce long-term side effects in a large number of patients, including chronic GVHD, tissue/organ dysfunction, abnormal immune response-related infections, recurrence and secondary cancers, and final patient quality of life [112,113]. In fact, the survival extension is no longer the sole goal of the treatment, but it should include the complete recovery of health, as well as social relationships [113]. To improve the outcomes, clinical studies should concern about HSC source for transplantation. It is recognized that the use of related umbilical cord blood could reduce the incidence and severity of chronic GVHD [114]. Interestingly, numerous clinical trials have revealed the effectiveness of MSC co-transplantation on controlling chronic GVHD, as well as other side effects related to HSC transplantation.

### 5.3. Drug Toxicity and Drug Resistance

The efficacy of using stem cells as gene and drug delivery carriers definitely depends on the number of cells localized within the tumor. In fact, only ~2–5% of total stem cells were reached to tumor tissue after their systemic injection, which was stable over time of observation [115,116]. Most of the intravenously injected cells were initially entrapped in lung parenchyma, then egressed to liver, spleen and lymph nodes, and finally cleared out from the body over time [115,117]. Therefore, this could raise some issues. First, the levels of non-targeted therapeutics are high sufficient to induce toxicity to normal tissues and organs. Secondly, insufficient local levels of drugs in tumors not only reduce the treatment efficacy, but also increase the risk for drug resistance [118,119]. Finding out the methods to improve targeting of transplanted stem cells to tumors may eliminate these risks and make this strategy reliable in clinical settings.

### 5.4. Increased Immune Responses and Autoimmunity

The application of allogeneic stem cells derived from unrelated donors may elicit severe host immune responses [120]. In fact, strong evidence has shown the formation of both specific cellular T-cell responses and B-cell mediated humoral antibodies targeting donor antigens [121,122,123,124]. Though it is found safe after the initial injection of these cells to patients of certain diseases, the pre-existing anti-donor memory responses would damage the next transplant and result in graft rejection [122,125].

In another aspect, the risk of autoimmunity would occur with the use of autologous iPSC-based whole-cell vaccines. These vaccines contain both CSC-specific and normal tissue-associated antigens. Therefore, it is possible to induce immune responses toward normal tissues. Nigel et al. did not observe autoimmunity after treatment with autologous iPSC vaccine [11]; however, further investigation needs to be carried out to confirm the safety of the approach.

### 5.5. Viral Infection

Viral transfection is a common and effective method to modify stem cells for gene delivery carriers. However, it possibly introduces the chance for a viral infection to recipients. The main associated problems are viral strong immunogenicity that could elicit adverse immune responses, causing toxin release, elimination of transduced cells, limited transgenic capacity size, and even death [126]. Therefore, viral vectors should be carefully modified to delete specific sequences that involve in inherent toxicity in patients while introducing targeted sequences for anti-cancer effect [126]. In addition, extensive preclinical evaluation is essential to confirm the safety and efficiency of viral vectors prior to translating the therapy into clinical setting.

## 6. Conclusions and Future Direction

In this review, we emphasize the biological roles of stem cells and update their diverse applications, as well as their side effects/risks in the treatment of cancers. Various stem cell types have been utilized for anticancer therapy, depending on their intrinsic capacities. HSC transplantation has provided an effective procedure for the treatment of hematologic cancers like leukemia, multiple myeloma, and lymphomas. However, severe GVHD found in some patients needs to be solved. The co-infusion of immunomodulatory MSCs exhibited high effectiveness in reducing GVHD cases, as well as on repairing injured tissues after heavy chemotherapy or radiotherapy, as evidenced by numerous clinical trials. On another aspect, MSCs and NSCs, as well as their exosomes, with intrinsic tumor-tropic properties, have extensively investigated to carry therapeutic materials toward tumors in preclinical models. However, “suicide gene therapy” has been the only application of stem cell carriers that are actively registered to the Phase I/II clinical trials till now. The most challenges are therapeutic dose control, low cell targeting and retention in tumor sites, which should be addressed in future studies. Moreover, induction of effector CAR immune cells from stem cells (i.e., iPSCs and HSCs) has been recently emerged. The defined induction protocol enables to produce large numbers of universal clinical-grade immune cells for further evaluation in man in the near future. Finally, targeting CSCs by delivery of anti-cancer vaccines derived from CSCs’ and PSCs’ antigens may provide promising strategies to prevent tumor growth, metastasis, and recurrence.

Despite success in both preclinical and clinical models, many challenges of stem cell therapy need to be overcome. Further experiments would be performed to shed light on the signaling of stem cells on tumor growth and metastasis in specific circumstances, thereby choosing a suitable strategy to engineer stem cells. In addition, given the complexity and immunosuppressive properties of the solid tumor microenvironment, combination with other therapies, such as immune checkpoint inhibitors (i.e., CTLA-4 and PD-1 antibodies), may offer better efficacy to eliminate cancer and its recurrence. For example, Quanyin Hu et al. engineered the surface of HSCs with PD-1 antibodies-decorated platelets for the treatment of recurrent leukemia in mice [127]. After intravenous injection, these HSCs rapidly migrated to BM to enable PD-1 antibodies release due to locally inflammatory tumor microenvironment. This significantly augmented the activated T cell responses to tumor cells and improved mice survival time [127]. On the other aspects, the procedure for the production of exosomes as well as anti-tumor vaccines needs to be standardized to achieve stable effects. In those contexts, optimization of cell culture, doses, and administration schedules are highly important. In summary, existing results from stem cell technologies are highly encouraging for tumor treatment but it still needs further efforts to improve the safety and efficacy before they could enter clinical trials.

## Figures and Tables

**Table 1:** Selected clinical trials testing the effectiveness of MSCs on reducing GVHD caused by allogeneic HSC transplantation in cancer patients.

Disease & Interventions	Phase	Clinical Outcome	NCT/Ref.
Recent Published/Completed Trials			
- Refractory chronic GVHD - Adipose tissue-MSCs (1–3 × 10^6^/kg) & cyclosporine, prednisone	I/II	71.4% patients alive, 80% patients achieved complete remission (CR) 100% patients were free of steroids at week 56 No side events related to MSC treatment	[54]
- Steroid-refractory grade III or IV acute GVHD - 72–100 × 10^6^ MSCs	II/III	At 24 weeks (primary endpoint), 12/25 (48%) patients achieved CR At 52 weeks, 48% patients receiving MSCs were alive No side events related to MSC treatment	[55]
- Refractory acute GVHD - Bone marrow-MSCs (2 × 10^6^ cells/kg), 3 doses for a week	I	5/7 patients achieved CR with remarkable decrease in inflammatory cytokines Good correlation between clinical responses with decrease in the level of biomarkers (Elafin, CK18, and Reg 3α)	[56]
- Refractory chronic GVHD - MSCs & mycophenolate mofetil (15 mg/kg × 3 doses/day × 42 days) & tacrolimus (0.06 mg/kg × 2 doses/day × 180 days)	II	At 100 days (primary endpoint), < 35% nonrelapse mortality (NRM) 19/20 patients achieved sustained engraftment of HSCs At 1 year, 10% NRM, 30% relapse, 80% overall survival, 60% non-relapse survival	NCT00504803/ [57]
- Sclerodermatous GVHD - Bone marrow-MSCs (10–20 × 10^6^ cells, infusion into anterosuperior iliac spine)	I	Reduce symptoms in all 4 patients. Dramatic increase in Th1/Th2 cell ratio No side events related to MSC treatment	[58]
Ongoing Trial			
Mesenchymoangioblast derived MSCs	I	Ongoing	NCT02923375

**Table 2:** Selected clinical trials of using stem cells as therapeutic carriers to treat cancers.

Stem Cell Therapy	Drug(s)	Tumor Type	Phase	Trial Number
CD-expressing NSCs	5-FC	Recurrent high-grade gliomas	Phase I, completed	NCT01172964
HSV-TK-expressing MSCs	Ganciclovir	Advanced, recurrent or metastatic gastrointestinal adenocarcinoma	Phase I/II, completed	EudraCT 2012-003741-15
CD-expressing NSCs	5-FC and leucovorin	Recurrent high-grade gliomas	Phase I, ongoing	NCT02015819
INF-β-expressing MSCs	-	Ovarian cancer	Phase I, completed	NCT02530047
TRAIL-expressing MSCs	-	Adenocarcinoma of lung	Phase I/II, recruiting	NCT03298763
ICOVIR5-infected MSCs	-	Metastatic and refractory solid tumors	Phase I/II, completed	NCT01844661
OMV-infected MSCs	-	Recurrent ovarian cancer	Phase I/II, recruiting	NCT02068794
Ad5-DNX-2401-infected MSCs	-	Recurrent high-grade glioma	Phase I, recruiting	NCT03896568

## Abbreviations

CXCR	CXC-chemokine receptor
SDF-1	stromal cell-derived factor 1
ATP	adenosine triphosphate
UTP	uridine triphosphate
LFA-1	lymphocyte function-associated antigen 1
VLA-4/5	very late antigen-4/5
CD44	cluster of differentiation 44
MMP-2/9	matrix metalloproteinase-2/9
ECM	extracellular matrix
CCL-25	C-C motif 25
IL	interleukin
TNF-α	tumor necrosis factor α
TGF	transforming growth factor
IDO	indoleamine 2,3-dioxygenase
iNOS	induced nitric oxide synthase
HLA-G	histocompatibility antigen type G
TSG-6	TNF-α stimulated gene-6
PGE-2	prostaglandin E2
STC-1	stanniocalcin-1
VEGF	vascular endothelial growth factor
HO-1	heme oxygenase-1
IGF-1	insulin-like growth factor-1
PI3K	phosphoinositide 3-kinase
PTEN	phosphatase and tensin homolog
JAK/STAT	Janus kinase/signal transducers and activators of transcription
NF-κB	nuclear factor kappa-light-chain-enhancer of activated B cells
Lgf5	leucine-rich repeat-containing G-protein coupled receptor 5
EpCAM	epithelial cell adhesion molecule
IFN	interferon
PLA	poly lactic acid
PLGA	poly (lactic-co-glycolic) acid
